# Supplementary material for: Causal Relationship between Aging and Anorexia Nervosa: A White-Matter-Microstructure-Mediated Mendelian Randomization Analysis
Source: Biomedicines. 2024 Aug 16;12(8):1874. doi: 10.3390/biomedicines12081874 (PMC11351342; doi:10.3390/biomedicines12081874)
Supplement: Supplementary file 1 [file biomedicines-12-01874-s001.zip › Supplementary Figure/Figure S4/Singe SNP plot of MR analysis for TL to ubm-b-1515.pdf]

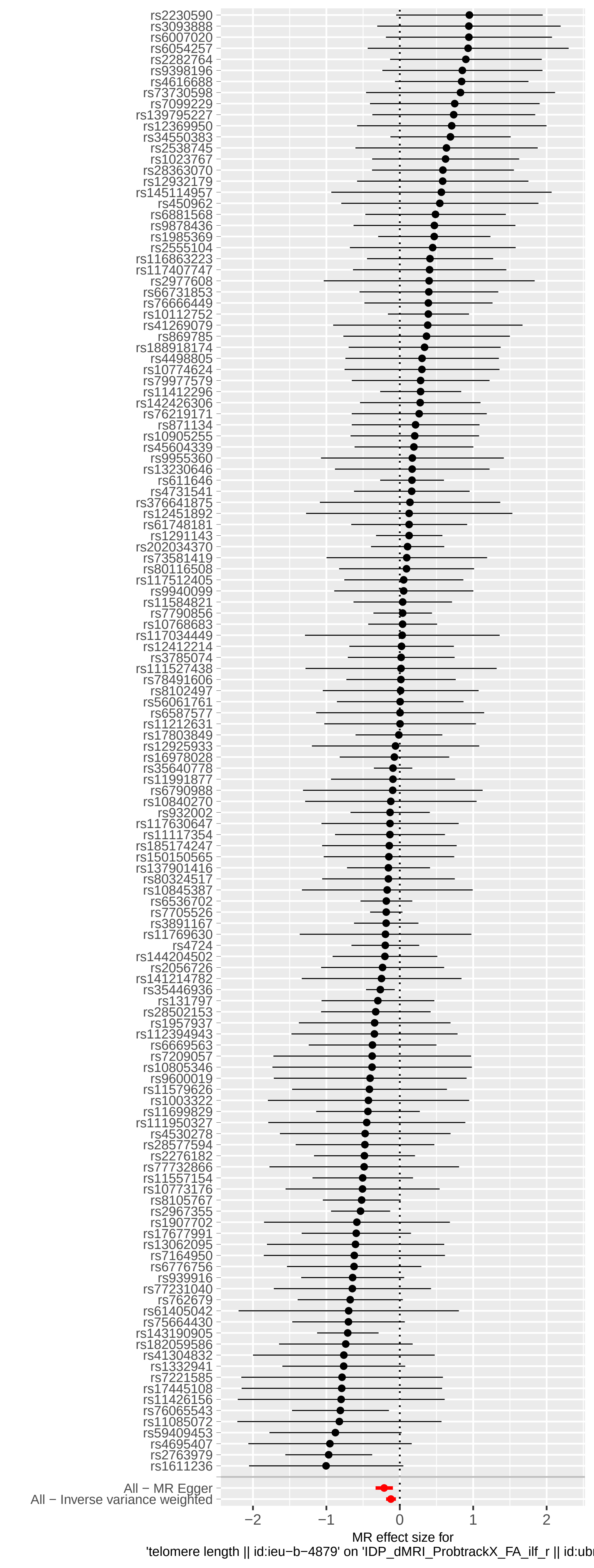
rs2230590
rs3093888
rs6007020
rs6054257
rs2282764
rs9398196
rs4616688
rs73730598
rs7099229
rs139795227
rs12369950
rs34550383
rs2538745
rs1023767
rs28363070
rs12932179
rs145114957
rs450962
rs6881568
rs9878436
rs1985369
rs2555104
rs116863223
rs117407747
rs2977608
rs66731853
rs76666449
rs10112752
rs41269079
rs869785
rs188918174
rs4498805
rs10774624
rs79977579
rs11412296
rs142426306
rs76219171
rs871134
rs10905255
rs45604339
rs9955360
rs13230646
rs611646
rs4731541
rs376641875
rs12451892
rs61748181
rs1291143
rs202034370
rs73581419
rs80116508
rs117512405
rs9940099
rs11584821
rs7790856
rs10768683
rs117034449
rs12412214
rs3785074
rs111527438
rs78491606
rs8102497
rs56061761
rs6587577
rs11212631
rs17803849
rs12925933
rs16978028
rs35640778
rs11991877
rs6790988
rs10840270
rs932002
rs117630647
rs11117354
rs185174247
rs150150565
rs137901416
rs80324517
rs10845387
rs6536702
rs7705526
rs3891167
rs11769630
rs4724
rs144204502
rs2056726
rs141214782
rs35446936
rs131797
rs28502153
rs1957937
rs112394943
rs6669563
rs7209057
rs10805346
rs9600019
rs11579626
rs1003322
rs11699829
rs111950327
rs4530278
rs28577594
rs2276182
rs77732866
rs11557154
rs10773176
rs8105767
rs2967355
rs1907702
rs17677991
rs13062095
rs7164950
rs6776756
rs939916
rs77231040
rs762679
rs61405042
rs75664430
rs143190905
rs182059586
rs41304832
rs1332941
rs7221585
rs17445108
rs11426156
rs76065543
rs11085072
rs59409453
rs4695407
rs2763979
rs1611236
All − MR Egger
All − Inverse variance weighted
−2
−1
0
1
2
MR effect size for
'telomere length || id:ieu−b−4879' on 'IDP_dMRI_ProbtrackX_FA_ilf_r || id:ub
